# Prehistoric Dentistry

**Published:** 1885-01

**Authors:** 


### PREHISTORIC DENTISTRY.

In the leading article of this number will be found an account of recent discoveries in Italy that are of great interest, not only to the dentist, but to the archæologist as well. The origin of the ancient Etruscan race is lost in the dimness of antiquity; but it was undoubtedly either Pelasgian or Lydian, and the probable date of their settlement in Italy was about one thousand years B. C. They were a powerful people when Romulus founded Rome, and very many of the laws and customs, the civic and military methods of organization of the latter people, were derived from the Etrurians. For many years Rome was engaged in wars with one or more of the twelve cities of Etruria, and the latter history of the one and the early history of the other are inextricably blended. Coriolanus, when banished from Rome, fled to Volci, one of the twelve cities of Etruria, and being placed in command of their forces had nearly captured his native city. Shakespeare's tragedy is founded upon this incident. Etruria was finally entirely subjugated to Rome, and the nation was amalgamated with the Romans.

The ancient Etrurians were a very remarkable people. Among them the finer mechanical arts were cultivated to a degree that excites the astonishment of even this age. They were luxurious in their habits and dress, and exceedingly fond of personal ornamentation. Etruscan jewelry has been famous for twenty-five hundred years. We have seen specimens of it more than two thousand years old, that would be difficult of reproduction to-day by any but the most skillful of artificers. Everything pertaining to their history is lost, save that which has been found in and about their wondrous tombs, of one of which Herodotus gives an account (I., 93). It is here that the archæologist would most naturally look for any fine gold work, and Dr. Van Marter has probably struck one of the richest veins for the archæological dentist that the world affords.

We are well aware that Herodotus speaks of dentists among the early Egyptians, and that travelers have presented accounts of specimens of dentistry and instances of teeth filled with gold among various peoples, some of whom were scarcely above barbarians in social status. But we also know that it was customary with some of the ancient nations to ornament the bodies of the dead by gilding the teeth, and inserting bits of copper and gold in the mouth. The early Romans, indeed, always placed a coin in the mouth of the dead, to pay Charon for ferriage across the river Styx. Inasmuch as every account of prehistoric dentistry had come from those who were not experts, and who had not sufficient of technical knowledge always to discriminate between ornamentation of the dead and veritable prosthetic operations, we had entertained the idea that such accounts were unreliable, and that ancient dentistry was a myth.

Dr. Van Marter is, however, competent authority, and he has established beyond question the existence of skilled dentists during prehistoric times. It might not be unprofitable for those who are accustomed to boast of modern achievements, especially in prosthetic dentistry, after examining the plates and reading the article of Dr. Van Marter, to again read Dr. Bristol's "Personal Recollections" in the December number, and learn how the artificial teeth of sixty years ago compared with those of six hundred before Christ. It is the good fortune of the INDEPENDENT PRACTITIONER to present, so far as we know, the first *fully attested* account of such work.

Dr. Van Marter writes us that during his absence in Germany the past summer, some workmen near Bologna dug up about a dozen specimens of artificial dentures, and sold them for old gold. He has taken measures to secure all such in the future, and armed with permission from the authorities, from the Pope as well as from the civil power, he proposes to continue his investigations, and has promised the INDEPENDENT PRACTITIONER the results of his enquiries. They will be looked for with interest.

